# A multi-country survey on access to healthcare and treatment services among individuals with critical medical care needs during the first wave of the pandemic

**DOI:** 10.1186/s12889-023-15007-0

**Published:** 2023-01-12

**Authors:** Morenike Oluwatoyin Folayan, Roberto Ariel Abeldaño Zuñiga, Jorma I. Virtanen, Oliver C. Ezechi, Muhammad Abrar Yousaf, Ala’a B. Al-Tammemi, Mohammed Jafer, Passent Ellakany, Eshrat Ara, Martin Amogre Ayanore, Anthonia Omotola Ishabiyi, Balgis Gaffar, Nourhan M. Aly, Joanne Lusher, Maha El Tantawi, Annie L. Nguyen

**Affiliations:** 1 Mental Health and Wellness Study Group, Ile-Ife, Nigeria; 2 Department of Child Dental Health, Obafemi Awolowo University, Ile-Ife, Nigeria; 3 Postgraduate Department, University of Sierra Sur, Oaxaca, Mexico; 4 Faculty of Medicine, University of Turku, Turku, Finland; 5 The Centre for Reproductive and Population Health Studies, Nigerian Institute of Medical Research, Yaba, Lagos, Nigeria; 6 Department of Biology, Faculty of Science and Technology, Virtual University of Pakistan, Lahore, Pakistan; 7 Applied Science Research Center, Applied Science Private University, Amman, Jordan; 8 Migration Health Division, International Organization for Migration (IOM), Amman, Jordan; 9 Faculty of Dentistry, Dental Public Health Division, Jazan University, Jizan, Saudi Arabia; 10 Department of Substitutive Dental Sciences, College of Dentistry, Imam Abdulrahman Bin Faisal University, Dammam, Saudi Arabia; 11 Department of Psychology, Government College for Women, J&K, MA Road Srinagar, Srinagar, India; 12 Department of Health Policy Planning and Management, Fred N. Binka School of Public Health, University of Health and Allied Sciences, Ho, Ghana; 13 Department of Sociology, Florida Atlantic University, Florida, USA; 14 Department of Preventive Dental Sciences, College of Dentistry, Imam Abdulrahman Bin Faisal University, Dammam, Saudi Arabia; 15 Department of Pediatric Dentistry and Dental Public Health, Faculty of Dentistry, Alexandria University, Alexandria, Egypt; 16 Provost’s Group, Regent’s University London, London, UK; 17 Department of Family Medicine, Keck School of Medicine, University of Southern California, Los Angeles, CA USA

**Keywords:** Access to healthcare, Alternative healthcare, Access to medicines, Sex worker, Drug use

## Abstract

**Background:**

Healthcare services were significantly interrupted during the early phase of the COVID-19 pandemic. The aim of the present study was to determine the associations between sociodemographic factors and healthcare access during the first wave of the COVID-19 pandemic among individuals with critical care needs.

**Methods:**

This was a secondary analysis of the data of 5,156 participants recruited from 152 countries during the first wave of the COVID-19 pandemic. The dependent variables were self-reported difficulty of access to health care, challenges with obtaining medication, and the use of alternative medical services. The independent variables were age at last birthday; sex at birth, level of education, employment status and the macro-social vulnerability status. The confounding variable was the country income level. Three multivariable logistic regression analyses were conducted to determine the associations between the dependent variables and the independent variables after adjusting for the confounder.

**Results:**

Difficulty accessing health care services and obtaining medications was experienced by 1922 (37.3%) and 3746 (72.7%) participants respectively. Also, 1433 (27.8%) used alternative medical care. Retirees (AOR:1.59), unemployed (AOR:1.198), people living with HIV (AOR:2.36) and at increased risk of COVID-19 (AOR:2.10), people who used drugs (AOR:1.83) and transacted sex (AOR:1.971) had significantly higher odds for reporting difficulty with access to health care. Males (AOR:1.23), respondents with secondary level of education (AOR:1.39), retirees (AOR:2.19), unemployed (AOR:1.47), people living with HIV (AOR:2.46), people who used drugs (AOR:1.79), transacted sex (AOR:2.71) and those who might be (AOR: 1.66) and were at (AOR: 2.3) increased risk of severe COVID-19 had significantly higher odds for reporting difficulty with access to medications. People who used drugs (AOR:2.093) transacted sex (AOR:1.639), who might be (AOR: 1.211) and were at (AOR: 1.511) increased risk of severe COVID-19, and who had difficulty accessing usual healthcare (AOR: 9.047) and obtaining medications (AOR:2.16) had significantly higher odds of reporting alternative medical care use. People living with HIV (AOR:0.562) had significantly lower odds of using alternative medical care.

**Conclusion:**

We identified populations who had challenges with access to healthcare and obtaining medications used alternative medical care except for people living with HIV. Priority attention should be given to alternative medical care use during future health pandemics.

## Introduction

Healthcare access disruption refers to difficulty with obtaining or using needed healthcare in proportion to the healthcare needs [1, 2]. The delivery of healthcare services was significantly disrupted during the early phase of the COVID-19 pandemic [3–6]. Restrictions on movement and business operation resulted in the disruption of access to routine and emergency communicable and non-communicable diseases hospital management [3]. The magnitude of the impact of the pandemic differed by population. For example, access of sexual minority women in Nigeria to sexual and reproductive health and HIV services was poorer when compared to other vulnerable women [7]. Services worse affected are long-term care for chronic conditions, rehabilitation, palliative end-of-life care [3] and HIV care for key HIV populations [8–10].

Populations whose access to healthcare must have also been disrupted during the pandemic are those who need a lot more hospital facility-based care such as females [11], people with lower educational status [12], elderlies [13] and populations that are macro-socially vulnerable like transactional sex workers and people who use drugs [14]. These are socio-economically disadvantaged populations who are also worse affected by the COVID-19 pandemic [15, 16]. These populations may more likely, seek alternative medical care.

Alternative medical care are medical modalities that are typically supported by tradition health care and not part of conventional medicine [17, 18]. The absence of effective COVID-19 pharmacotherapy led to a surge in the demand and supply of many alternative medical practices and care through pharmacists and healthcare staff some of which have potential benefits in COVID-19 patient and consumer behaviours [19]. These benefits include the enhancement of the immune system [20, 21] and the reduction in pandemic-related anxiety [22].

Many individuals may have resorted to the use of alternative medical care because of poor access to conventional healthcare facilities. Access to conventional healthcare facilities was poor due to the diversion of care focus to the large number of patients that needed emergency care for COVID-19 and poor use of health facilities due to concerns about contracting SARS-CoV-2 infection [23]. The countries’ income level may however, affect the formulation of policies and the strength of the health care systems to address the COVID‐19 pandemic. There is however, little known about how the pandemic drove the uptake and use of alternative medical care during the COVID-19 pandemic by those who had critical health needs and had challenges with access to routine health care facilities. The aims of this study were to determine whether access to healthcare services were disrupted for some populations more than others. Specifically, the study determined the associations between sociodemographic factors (age, sex, education status, employment status, macro-social vulnerability status) and healthcare access by individuals with critical care needs during the first wave of the COVID-19 pandemic. In addition, we determined if there was an association between reported disruption in access to healthcare services and the use of alternative medical care. We hypothesised that lower education and employment status and macro-social vulnerability status would have significantly associated with poorer access to healthcare services and increased use of alternative medical services.

## Methods

### Ethical considerations

Ethical approval was obtained from the Human Research Ethics Committee at the Institute of Public Health of the Obafemi Awolowo University Ile-Ife, Nigeria (HREC No: IPHOAU/12/1557). Additional ethical approvals were obtained from Brazil (CAAE N° 38,423,820.2.0000.0010), India (D-1791-uz and D-1790-uz), Saudi Arabia (CODJU-2006F) and the United Kingdom (13,283/10570) for the conduct of the primary study.

### Study design and study participants

This was a secondary analysis of data generated through a cross-sectional study that recruited 21,106 adults from 152 countries through an online survey platform (Survey Monkey, Momentive Inc.: San Mateo, CA, USA) between July and December 2020 during the first wave of the COVID-19 pandemic. Study participation was open to anyone 18 years and older. There were no exclusion criteria. Study participants checked a box to indicate their consent before participating in the online survey. Details of the study methodology and data collection tools are reported elsewhere [24–26].

### Sample size

The sample size for the primary study was calculated based on the highest global prevalence of a mental health disorder in 2019. The pre-survey minimum sample size for this study was set at 59 valid respondents from each of the 193 member States of the United Nations based on the estimated prevalence of anxiety disorder of 3.94% [27], a desired precision of estimate of 0.05 and a confidence level of 95% for an infinite population size [28]. From statistical modelling, a minimum of 10 participants with complete responses per independent variable enables the performance of regression analyses with a minimum probability level (*p*-value) of 0.05 [29].

For this study, complete data of 5,156 (24.4%) participants who self-identified as having critical medical needs were extracted from the dataset. Critical medical needs in this context, means *care that all critically ill patients should receive in all hospitals in the world* [30]. Respondents with critical medical needs were identified by those who responded ‘yes’ to the question: did you have critical medical need during the COVID-19 pandemic?

### Study participants recruitment

Study participants were recruited through respondent-driven sampling. Initial participants reached by 45 members of the MEHEWE Study Group (www.mehewe.org) were asked to share the survey link with their contacts around the world. The survey link was also posted on social media groups (Facebook, Twitter, and Instagram), network email lists and WhatsApp groups.

### Data collection tool

The overall content validity index for the study questionnaire was 0.83. The dimensionality and reliability of the tool were also assessed. Details on the validation of the data collection tool have also been published [24]. The instrument was developed in English and the validated tool further translated to French, Spanish, Arabic and Portuguese.

### Dependent variables

Disruption to healthcare services was determined by self-reported challenges with access to healthcare and challenges with obtaining medication. Use of alternative medical services was also determined by self-report. Respondents were asked: Did you have challenges accessing usual medical health care services (yes/no); did you have difficulty with obtaining medications (yes/no); and did you have to resort to alternative medical care services to address your health needs (yes/no)? These questions were adapted from the section of the Pandemic Stress Index that rated the impact of the COVID-19 experience on daily life. The content validity index of the Pandemic Stress Index was 0.90 [24].

### Independent variables

The independent variables included the socio-demographic profile of the respondents: age; sex at birth (male, female, others); level of education (no formal education, primary, secondary and college/university); employment status (retiree, student, unemployed, employed) and the macro-social vulnerability status. Macro-social vulnerability status was defined as HIV key populations (people who transacted sex, people who used drugs) and people living with HIV. All responses were based on self-report.

Respondents were categorised for risk for severe COVID-19 (little or no risk, might be at increased risk, or at increased risk) based on their medical conditions [30]. Respondents identified if they had a medical condition by checking from a list of medical conditions or naming their medical condition if not listed. The list was categorised into risk profile [31].

### Confounding variable

The confounding variable was country income level obtained from publicly available data of the 2019 World Bank Data Bank [32]. Based on the income level, countries were classified into low‐income countries (LICs) with a gross national income (GNI) per capita ≤ 1035 USD in 2019, lower middle‐income countries (LMICs) with GNI between 1036 and 4045 USD, upper middle‐income countries (UMICs) with GNI between 4046 and 12,535 USD, and high‐income countries (HICs) with GNI ≥ 12,536 USD.

### Data analysis

Raw data were downloaded, cleaned, and imported to SPSS version 23.0 (IBM Corp., Armonk, N.Y., USA) for analyses. Three multivariate logistic regression models were developed. Two adjusted multivariate logistic regression models were developed to determine the factors associated with difficulty accessing access to healthcare and challenges with obtaining medication. A third adjusted multivariate logistic regression analysis was conducted to determine the factors associated with the use of alternative medical services. This model included all independent variables added to the first two models and two additional variables: difficulty with access to health care services and challenges with obtaining medication. Adjusted odds ratios (AORs), 95% confidence intervals (CIs) and *p* values were calculated. Statistical significance was set at < 5%.

## Results

Table 1 shows that of the 5,156 participants with a critical care need, 1,922 (37.3%) had challenges with access to health care services and 1,410 (27.3%) had difficulty obtaining medications. A greater proportion of people with a primary level of education (46.8%), retirees (50.8%), living with HIV (53.6%), who used drugs (54.3%), who had transacted sex (58.8%), and were at increased risk for severe COVID-19 (56.2%) had challenges with access to health care services. Also, more males (29.9%), people with no formal education (45.5%), who were unemployed (34.5%), living with HIV (48.2%), used drugs (44.1%), transacted sex (56.0%) and who were at increased risk for severe COVID-19 (43.2%) had difficulty obtaining medications.

**Table 1:** Multivariate regression analysis of factors associated with difficulty with access to health care services and obtaining medications by people critically ill during the first wave of the COVID-19 pandemic (*N* = 5156)

Variables	Total *N* = 5156 n (%)	Challenges with access to health care			Difficulty with obtaining medication		
		Yes *N* = 1922 (37.3) n (%)	No *N* = 3234 (62.7) n (%)	AOR; 95% CI; *p* value	Yes *N* = 1410 (27.3%) n (%)	No *N* = 3746 (72.7%) n (%)	AOR; 95% CI; *p* value
**Age mean (SD)**	36.08 (12.5)	36.9 (12.7)	35.6 (12.4)	0.998; 0.992–1.004; *p* = 0.443	35.8 (12.7)	36.2 (12.4)	0.987; 0.980–0.993; *p* < 0.001
**Economic region**							
LICs	141 (2.7)	73 (51.8)	68 (48.2)	1.697; 1.183–2.434; *p* = 0.004	49 (34.8)	92 (65.2)	2.100; 1.425–3.094; *p* < 0.001
LMICs	2960 (57.4)	1006 (34.0)	1954 (66.0)	0.715; 0.610–0.837; *p* < 0.001	831 (28.1)	2129 (71.9)	1.186; 0.989–1.423; *p* = 0.066
UMICs	1045 (20.3)	427 (40.9)	618 (59.1)	1.073; 0.894–1.289; *p* = 0.449	287 (27.5)	758 (72.5)	1.407; 1.140–1.737; *p* = 0.001
HICs	1010 (19.6)	416 (41.2)	594 (58.8)	1.000	243 (24.1)	767 (75.9)	1.000
**Sex at birth**							
Male	2205 (42.8)	820 (37.2)	1385 (62.8)	0.954; 0.946–1.075; *p* = 0.441	659 (29.9)	1546 (70.1)	1.225; 1.075–1.396; *p* = 0.002
Female	2951 (57.2)	1102 (37.3)	1849 (62.7)	1.000	751 (25.4)	2200 (74.6)	1.000
**Level of education**							
No formal education	44 (0.9)	18 (40.9)	26 (59.1)	0.798; 0.423–1.505; *p* = 0.485	20 (45.5)	24 (54.5)	1.426; 0.757–2.683; *p* = 0.272
Primary	94 (1.8)	44 (46.8)	50 (53.2)	0.961; 0.621–1.487; *p* = 0.858	35 (37.2)	59 (62.8)	0.964; 0.611–1.522; *p* = 0.876
Secondary	875 (17.0)	343 (39.2)	532 (60.8)	1.000; 0.849–1.177; *p* = 0.996	312 (35.7)	563 (64.3)	1.392; 1.173–1.651; *p* < 0.001
College/University	4143 (80.4)	1517 (36.6)	2626 (63.4)	1.000	1043 (25.2)	3100 (74.8)	1.000
**Employment status**							
Retiree	179 (3.5)	91 (50.8)	88 (49.2)	1.592; 1.133–2.237; *p* = 0.007	68 (38.0)	111 (62.0)	2.191; 1.529–3.141; *p* < 0.001
Student	881 (17.1)	287 (32.6)	594 (67.4)	0.955; 0.791–1.153; *p* = 0.632	220 (25.0)	661 (75.0)	0.936; 0.761–1.153; *p* = 0.536
Unemployed	876 (17.0)	359 (41.0)	517 (59.0)	1.198; 1.020–1.407; *p* = 0.028	302 (34.5)	574 (65.5)	1.471; 1.239–1.747; *p* < 0.001
Employed	3220 (62.5)	1185 (36.8)	2035 (63.2)	1.000	820 (25.5)	2400 (74.5)	1.000
**Living with HIV**							
Yes	504 (9.8)	270 (53.6)	234 (46.4)	2.357; 1.913–2.904; *p* < 0.001	243 (48.2)	261 (51.8)	2.458; 1.984–3.045; *p* < 0.001
No	4652 (90.2)	1652 (35.5)	3000 (64.5)	1.000	1167 (25.1)	3485 (74.9)	1.000
**Use drugs**							
Yes	374 (7.3)	203 (54.3)	171 (45.7)	1.831; 1.463–2.291; *p* < 0.001	165 (44.1)	209 (55.9)	1.797; 1.428–2.262; *p* < 0.001
No	4782 (92.7)	1719 (35.9)	3063 (64.1)	1.000	1245 (26.0)	3537 (74.0)	1.000
**Transact sex**							
Yes	277 (5.4)	163 (58.8)	114 (41.2)	1.973; 1.516–2.567; *p* < 0.001	155 (56.0)	122 (44.0)	2.713; 2.079–3.539; *p* < 0.001
No	4879 (94.6)	1759 (36.1)	3120 (63.9)	1.000	1255 (25.7)	3624 (74.3)	1.000
**Risk for severe COVID-19**							
Might be at increased risk	939 (18.2)	419 (44.6)	520 (55.4)	1.481; 1.274–1.722; *p* < 0.001	321 (34.2)	618 (65.8)	1.658; 1.410–1.950; *p* < 0.001
At increased risk	345 (6.7)	194 (56.2)	151 (43.8)	2.102; 1.655–2.669; *p* < 0.001	149 (43.2)	196 (56.8)	2.321; 1.811–2.976; *p* < 0.001
Little or no risk	3872 (75.1)	1309 (33.8)	2563 (66.2)	1.000	940 (24.3)	2932 (75.7)	1.000

The odds of having challenges with access to healthcare were higher for retirees (AOR: 1.592; *p* = 0.007) and those unemployed (AOR: 1.198; *p* = 0.028) when compared to those who were employed. The odds were also higher for people living with HIV (AOR: 2.357; *p* < 0.001) compared to those not living with HIV and for people who used drugs (AOR: 1.831; *p* < 0.001) and transacted sex (AOR: 1.973; *p* < 0.001) compared to those who did not use drugs or transacted sex. Also, people at increased risk of severe COVID-19 (AOR: 2.102; *p* < 0.001) had significantly higher odds of having challenges with access to healthcare compared to those with little or no risk for severe COVID-19.

The odds of having challenges obtaining medications were higher for males compared to females (AOR:1.225; *p* = 0.002), those with secondary level education (AOR: 1.392; *p* < 0.001) compared to college/university level of education, retirees (AOR: 2.191; *p* < 0.001) and those unemployed (AOR: 1.471; *p* < 0.001) compared to those who were employed, people living with HIV (AOR: 2.458; *p* < 0.001) compared to those not living with HIV, people who used drugs (AOR: 1.797; *p* < 0.001) compared to people who did not, and people who transacted sex (AOR: 2.713; *p* < 0.001) when compared to those who did not. Also, respondents who might be at increased risk (AOR: 1.658; *p* < 0.001) and those who were at increased risk (AOR: 2.321; *p* < 0.001) of severe COVID-19 when compared to those with little or no risk for severe COVID-19; and those living in LICs (AOR: 2.100; *p* < 0.001) and UMICs (AOR: 1.407; *p* = 0.001) when compared to those living in HICs had significantly higher odds for reporting difficulty with access to medications. The odds of having challenges obtaining medications decreased with age (AOR: 0.987; *p* < 0.001).

Table 2 shows that out of the 5,156 participants with a critical care need, 1,433 (27.8%) used alternative medical care. More males (28%), people with primary level of education (42.6%), unemployed (28.8%), living with HIV (30%), used drugs (50.8%), transacted sex (50.2%), have little or no risk of severe COVID-19 (75.2%), had challenges accessing usual healthcare (56.7%), and had challenges obtaining medications (50.9%) used alternative medical care.

**Table 2:** Multivariate regression analysis of factors associated with the use of alternative medical services by people critically ill during the first wave of the COVID-19 pandemic (*N* = 5186)

Variables	Total *N* = 5156 n (%)	Used alternative medical services		AOR; 95% CI; *p* value
		Yes = 1433 (27.8%) n (%)	No = 3723 (72.2%) n (%)	
**Age mean (SD)**	36.08 (12.5)	35.6 (12.1)	36.2 (12.6)	0.994; 0.987–1.002; *p* = 0.127
**Economic region**				
LICs	141 (2.7)	64 (45.4)	77 (54.6)	2.121; 1.373–3.277; *p* = 0.001
LMICs	2960 (57.4)	791 (26.7)	2169 (73.3)	1.161; 0.950–1.419; *p* = 0.145
UMICs	1045 (20.3)	281 (26.9)	764 (73.1)	0.957; 0.756–1.210; *p* = 0.711
HICs	1010 (19.6)	297 (29.4)	713 (70.6)	1.000
**Sex at birth**				
Male	2205 (42.8)	617 (28.0)	1588 (72.0)	0.909; 0.783–1.057; *p* = 0.214
Female	2951 (57.2)	816 (27.7)	2135 (72.3)	1.000
**Level of education**				
None	44 (0.9)	17 (38.6)	27 (61.4)	1.649; 0.775–3.507; *p* = 0.194
Primary	94 (1.8)	40 (42.6)	54 (57.4)	1.843; 1.099–3.091; *p* = 0.020
Secondary	875 (17.0)	218 (24.9)	657 (75.1)	0.736; 0.598–0.906; *p* = 0.004
College/University	4143 (80.4)	1158 (28.0)	2985 (72.0)	1.000
**Employment status**				
Retiree	179 (3.5)	43 (24.0)	136 (76.0)	0.540; 0.345–0.845; *p* = 0.007
Student	881 (17.1)	235 (26.7)	646 (73.3)	1.066; 0.845–1.346; *p* = 0.590
Unemployed	876 (17.0)	252 (28.8)	624 (71.2)	0.939; 0.768–1.149; *p* = 0.544
Employed	3220 (62.5)	903 (28.0)	2317 (72.0)	1.000
**Living with HIV**				
Yes	504 (9.8)	151 (30.0)	353 (70.0)	0.562; 0.433–0.730; *p* < 0.001
No	4652 (90.2)	1282 (27.6)	3370 (72.4)	1.000
**Use drugs**				
Yes	374 (7.3)	190 (50.8)	184 (49.2)	2.093; 1.609–2.721; *p* < 0.001
No	4782 (92.7)	1243 (26.0)	3539 (74.0)	1.000
**Transact sex**				
Yes	277 (5.4)	139 (50.2)	138 (49.8)	1.639; 1.198–2.241; *p* = 0.002
No	4879 (94.6)	1294 (26.5)	3585 (73.5)	1.000
**Risk for severe COVID-19**				
Might be at increased risk	939 (18.2)	619 (65.9)	320 (34.1)	1.211; 1.005–1.459; *p* = 0.044
At increased risk	345 (6.7)	193 (55.9)	152 (44.1)	1.511; 1.134–20.13; *p* = 0.005
Little or no risk	3872 (75.1)	2911 (75.2)	961 (24.8)	1.000
**Challenges accessing usual medical health care**				
Yes	1922 (37.3)	1090 (56.7)	832 (43.3)	9.047; 7.744–10.571; *p* < 0.001
No	3234 (62.7)	343 (10.6)	2891 (89.4)	1.000
**Difficulty with obtaining medication**				
Yes	1410 (27.3)	718 (50.9)	692 (49.1)	2.164; 1.843–2.541; *p* < 0.001
No	3746 (72.7)	715 (19.1)	3031 (80.9)	1.000

*AOR* Adjusted odds ratio, *CI* Confidence interval

Significantly higher odds of reporting alternative medical care use was reported by people with primary level education compared to those with college/university education (AOR:1.843; *p* < 0.001), respondents who used drugs (AOR:2.093; *p* < 0.001) and transacted sex (AOR:1.639; *p* = 0.002) compared to those who did not, and those who might be at increased risk (AOR: 1.211; *p* = 0.044) and those at increased risk (AOR: 1.511; *p* = 0.005) of severe COVID-19 compared to those with little or no risk. Also, respondents who had difficulty accessing usual healthcare (AOR: 9.047; *p* < 0.001) compared to those who had no difficulty, those who had challenges obtaining medications (AOR:2.164; *p* < 0.001) compared to those who had no challenges and those living in LIC compared to those living in HIC (AOR: 2.121; *p* = 0.001) had significantly higher odds of reporting alternative medical care use. Respondents with secondary school education (AOR: 0.736; *p* = 0.004), retirees (AOR: 0.540; *p* = 0.007) and people living with HIV (AOR:0.562; *p* < 0.001) had significantly lower odds of using alternative medical care.

## Discussion

The study findings showed that employment status, the risk for severe COVID-19 infection and macro-social vulnerability status may be risk factors for challenges with access to health care, obtaining medications, and the use of alternative medical services by persons who had critical care needs during the first wave of the COVID-19 pandemic. Age, sex and educational status may also be risk factors for medications challenges whereby younger age, males and those with a secondary level of education may be more likely to have experienced difficulty accessing medications. Employment status was also associated with the use of alternative health services. In addition, people who had challenges accessing usual healthcare, difficulty with obtaining medications and those who had a primary level of education seem more likely to have used alternative medical services. People with secondary level education seem were less likely to have used alternative medical services relative to those with college/university education. The study findings supported the study hypothesis that socially disadvantaged groups will face more challenges accessing healthcare, obtaining medications, and using alternative medical healthcare services during the first wave of the COVID-19 pandemic.

Though it is well recognised that the COVID-19 pandemic posed a global challenge for the provision of healthcare services and its accessibility during the first wave [33], little is known about how it drove inequalities in healthcare access for different populations [34]. To our knowledge, this study is one of the few studies to highlight the impact of the pandemic on inequalities in access to care among groups of populations with specific demographics. Moreover, it sheds light on the factors influencing the use of alternative health services. The large sample size also enabled the conduct of sub-group analysis.

Limitations of this study include the cross-sectional design, which limits the ability to establish causal-effect relationships between the study variables. The use of online data collection excluded people who had no access to internet and smartphones. This introduces selection bias to the study and limits the generalisability of results. Online data collection and the use of non-probability sampling were, however, the options available for global data collection during the first wave of the pandemic when movement and contact were restricted in most countries [35] though this method was associated with lower response rates [36]. The self-reporting of HIV status that is stigmatised by many communities may also result in under-reporting [37]. Despite these limitations, the study findings provide insight relevant to improving the healthcare delivery system for specific populations who are in critical need of healthcare services during major crisis like the COVID-19 pandemic.

We identified that people living with HIV, people who use drugs, people who had transactional sex and people who might be or were at increased risk for COVID-19 had challenges accessing health care and accessing medications. Multiple factors contribute to the poor access of those in need of critical care including the diversion of resources to address the urgent needs associated with the COVID-19 pandemic [38]. At the individual level, reduced income might have also affected access to care [39, 40]. Other barriers to health care access include decreased use of healthcare services due to country measures on elective care, fear of contagion and stigma; low health literacy [23, 41], and inability to access services due to low socioeconomic status and poor competency to use technologies [35]. Prior studies indicated that people with needs for antiretroviral therapy [42], sexual and reproductive health services [43] and tuberculosis services [7, 44] among others, had difficulty accessing services during the COVID-19 pandemic.

For the macro-socially vulnerable populations, the use of alternative care seems to have helped address the gap in access to conventional healthcare created by the pandemic. Macro-socially vulnerable populations had challenges with access to medical care prior to the pandemic. The challenges to access to care by the population were created by widespread stigma and discrimination, restrictive laws and policies, state and non-state violence, harassment and criminalisation of behaviours or practices [45]. It is therefore possible that during the pandemic, the use of alternative medical healthcare services was an easy alternative choice to make for macro-socially vulnerable populations as they may have had prior understanding of how to navigate these spaces to access healthcare. However, for those who required care for chronic conditions in areas without well-established alternative healthcare services, the pandemic may have worsened their access to healthcare services [46]. Also, people may have opted for alternative care because of the fear of contagion, a reason that kept many away from the health facilities [47].

People who had HIV were less likely to use alternative medical care. Active advocacy for the care and welfare of people living with HIV during the pandemic was the precursor to design many interventions tailored for the population having or at risk of contracting HIV in many countries. Consequentially, it enhances their access to healthcare through specially organized alternative care routes including e-health programs [48, 49]. People living with HIV may have therefore, found it less challenging to access their health care needs and therefore, resorted less to alternative medical care.

The findings of the present study indicate that there may have been some populations in critical need of medical care that were left behind during the first wave of the pandemic. These include younger people, males and those with secondary level of education who had difficulty in accessing necessary medications for their critical health conditions. These findings had been reported in a prior study [50]. People with secondary level of education were also less likely to have used an alternative healthcare. However, more studies are needed to elaborate on this finding.

Finally, our study findings suggest that the country of residence may be a moderating factor for access to health care, medications and access to alternative medical care services. We observed that people who reside in LICs may not only had challenges accessing healthcare and obtaining medications [51, 52] but also seem more likely not be able to use alternative medical care services. Though people in HICs had challenges with accessibility to healthcare services during the COVID-19 pandemic [32] the study results indicates that they had less challenges with access to healthcare when compared with LICs and UMICs; obtaining medications when compared with LICs, LMICs and UMICs; and use of alternative medical care when compared with LICs and LMICs during the first wave of the COVID-19 pandemic. It is possible that HICs with cutting-edge technologies, high availability of healthcare facilities and a reasonable number of healthcare professionals made it easier for citizens to have ready access to alternative medical care during the pandemic in ways which was less feasible for people in low resource settings [53, 54].

Paradoxically, though people in UMICs – a region with healthcare profile close to that of HICs—had more challenges in accessing healthcare and obtaining medications when compared to people in HICs, they seem to have less challenges with the use of alternative medical care. The use of alternative medicines seems to be more popular in middle-income countries howbeit that its use in these countries is overestimated [55]. Similarly, people with critical care need in LMICs seem to had less challenges with access to healthcare than those in HICs during the pandemic. This may be because the COVID-19 pandemic health-related crisis was less critical in this region than it was HICs and thus, the crowding out of people from the traditional healthcare system was less [56]. There is also the view that the income categorisation of countries is a false representation of their global health response capacity [57]. Future studies are needed to explore the observed income country differences in healthcare access during the pandemic.

In conclusion, this study determined that some socio-demographic factors influenced access to healthcare service, medications and use of alternative medical services during the first wave of the COVID-19 pandemic. These study findings suggest that the impact of the pandemic on access to care at the different healthcare delivery at different points in time, is a complex one, and populations vulnerable to poor healthcare access are worse affected. Those who faced challenges with accessing routine healthcare services and obtaining medications seem to have resorted to alternative health care, but little is known about how the uptake of alternative health care addressed their health care needs. This gap in number needs to be addressed in future studies.

## Data Availability

The datasets used and/or analysed during the current study are available from the corresponding author on reasonable request.

## Abbreviations

AOR: Adjusted Odds Ratio
CI: Confidence Interval
COVID-19: Coronavirus infectious disease 2019
GNI: Gross national income
HICs: High‐income countries
LICs: Low‐income countries (LICs)
LMICs: Lower middle‐income countries
SD: Standard Deviation
UMICs: Upper middle‐income countries
